# Jaguar Attack on a Child: Case Report and Literature Review

**DOI:** 10.5811/westjem.2015.1.24043

**Published:** 2015-02-26

**Authors:** Kenneth V. Iserson, Adama M. Francis

**Affiliations:** * The University of Arizona, Department of Emergency Medicine, Tucson, Arizona; † Georgetown Public Hospital (GPHC), Department of Emergency Medicine, Georgetown, Guyana

## Abstract

Jaguar attacks on humans rarely occur in the wild. When they do, they are often fatal. We describe a jaguar attack on a three-year-old girl near her home deep in a remote area of the Guyanese jungle. The patient had a complex but, relatively, rapid transport to a medical treatment facility for her life-threatening injuries. The child, who suffered typical jaguar-inflicted injury patterns and survived, is highlighted. We review jaguar anatomy, environmental status, hunting and killing behaviors, and discuss optimal medical management, given the resource-limited treatment environment of this international emergency medicine case.

## INTRODUCTION

Worldwide, hundreds of deaths are caused by large cat attacks annually.^1^ Reported attacks are rare in the Western Hemisphere or Europe, and usually occur in zoos and circuses, or are caused by “exotic” pets,^2^ especially where local laws and regulations are more lenient.^3^ Attacks on humans remain common in Africa and Asia, where significant wild populations of large cats still exist. They have become especially prevalent in areas where expansion of urban centers and agricultural zones have decreased these animals’ habitat size, reduced their natural prey, and forced them to hunt outside of their protected areas.^4^

**Figure 1:**
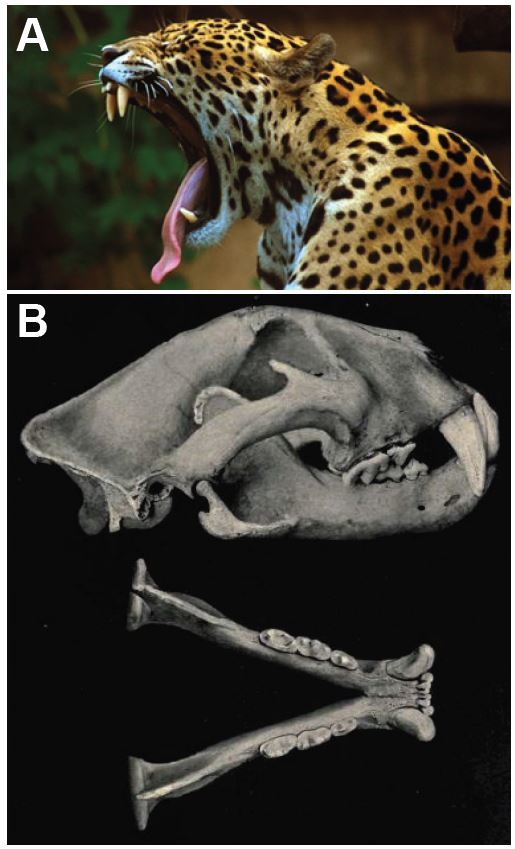
*A*, Jaguar (used by permission; *Panthera onca*, jaguar © MarcusObal, Creative Commons Attribution-Share Alike 3.0 Unported. Available at: http://www.nhm.ac.uk/nature-online/species-of-the-day/biodiversity/endangered-species/panthera-onca/biology/index.html. Accessed 6 July 2014.) *B,* Jaguar Skull (Public domain. Originally from Elliot DG. *The land and sea mammals of Middle America and the West Indies*. Chicago:Field Colombian Museum, 1904. Available at: http://commons.wikimedia.org/wiki/File:Jaguarskull.jpg#mediaviewer/File:Jaguarskull.jpg. Accessed 6 July 2014.)

Jaguars (*Panthera onca*) are the third largest felid (cat) after the tiger and the lion (Figures 1A and 1B). They exist only in the Western Hemisphere, with the Amazon regions of South America, particularly Brazil, having the highest concentration. An apex predator (no natural enemies), jaguar survival is threatened only when humans intentionally kill them or decrease their ability to feed by destroying or encroaching on their habitat. In some areas, their fear of humans has decreased due to ecotourism and intentional feeding. As in the following case of an unprovoked attack on a three-year-old Amerindian girl in Guyana, the cats may be more prone to attack humans as prey.

### Case Report

A three-year-old Amerindian girl presented without warning to our emergency department (ED) in the country’s only tertiary care hospital, after being attacked by a jaguar in the remote Isseneru Village, Cuyuni-Mazaruni (Region 7), Guyana. This village is located near the large Mazaruni River, amid dense jungle about 40 air miles from the Venezuelan border. When attacked, she was with her mother at the Mazaruni River, as they were every morning, to bathe and wash clothes.

Her mother says that she turned away from the child to tend to the clothes when she heard “screams and then saw the jaguar ripping away at her” (personal communication from Dr. Sagon, the area general medical officer, who visits that area every 2 to 3 months). The large adult jaguar apparently pounced on the girl from the bushes and, with its jaws clamped on her head, dragged the 24kg child about 60 feet into nearby bushes. It released her only after other residents, who had heard her screams, forced the jaguar to drop the child and then shot the animal.^5^

Although jaguar sightings in the region are rare, the child’s grandmother had witnessed that same jaguar attack the girl one month previously when they were at the river, “the animal jumped on her and scraped her on her foot. But I hit it and it got away.”^5^

The second attack, which caused grievous injuries, occurred at about 10:30am, and relatives rushed the child to the local health center by speedboat, about 10 minutes away. Thinking that the child was already dead, the family left her at the river bank and ran to get the health center nurse. When the nurse got to the child, she found a pulse, so the relatives waited about 10 minutes for a river boat to take them to the nearest radio so they could call a medical evacuation plane. The village and surrounding area has no phone service. Meanwhile, the nurse administered 200mL Ringers Lactate and 500mg metronidazole intravenously (IV) and dressed the wounds, although shortly thereafter, the agitated child pulled out her IV line and pulled off her dressings. The flight was quickly arranged, but it took about an hour to transport the child, via speedboat, to the airstrip. The child then had a 1½ hour flight to Georgetown, followed by a 15 minute ambulance transfer to our hospital.

**Figure 2:**
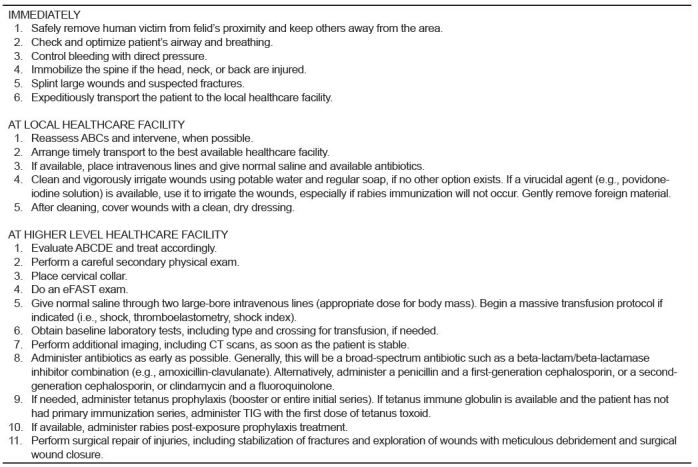
Optimal actions, evaluation and treatment for large felid injuries.^1^,^3^,^16^,^21^ *eFAST*, extended focused assessment with sonography for trauma; *ABC;* Airway,Breathing, Circulation; *ABCDE*, Airway, Breathing, Circulation, Disability, Exposure; *CT*, computed tomography; *TIG*, tetanus immune globin

The child arrived at our ED approximately five hours following the attack. Adhering as closely as possible to the optimal treatment regimen given the available resources (Figure 2), the patient was immediately taken to the ED critical treatment area, where her vital signs were blood pressure 62/42mmHg, pulse 102/minute, temperature 99.3°F axillary, and respiratory rate 18/minute. Her random blood sugar was 258mg/dL. The pulse oximeter was not functioning that day.

**Figure 3:**
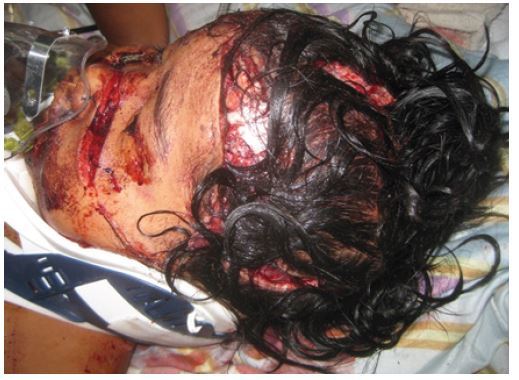
Child in the emergency department.

The ED staff performed a rapid physical exam, quickly placed the child in a cervical collar, cleaned and dressed her wounds, and started two peripheral IV lines (Figure 3). They administered ceftriaxone 1 gram, metronidazole 180mg, and 2 liters of Ringers Lactate. Laboratory results on admission were hemoglobin (Hgb) 5.0g/dL, white blood cell (WBC) 16,500/mm^3^ (Polys 73%, Lymphs 25%, Eos 1%, Bands 1%), platelets 364,000/μL, Na 139mEq/L, K 3.6mEq/L, Cl 108mEq/L.

Physical examination showed an awake, alert and cooperative child with multiple deep lacerations over her scalp, face, and torso. A puncture of the skull was observed within a scalp laceration. A portable chest radiograph and an eFAST exam demonstrated no abnormalities. At that point, general, orthopedic, and neurosurgery consults were obtained. Ophthalmology became involved near the end of the patient’s hospital stay.

A computed tomography (CT) without contrast was performed at a hospital 10 minutes away because the local CT scanner was inoperative. It demonstrated fractures along the left frontal bone involving the superior border of the left orbit, small areas of pneumocephalus in the cortical and interhemispheric regions, non-hemorrhagic contusion/edema in the left frontal lobe, and soft-tissue swelling of the scalp and face.

**Figure 4:**
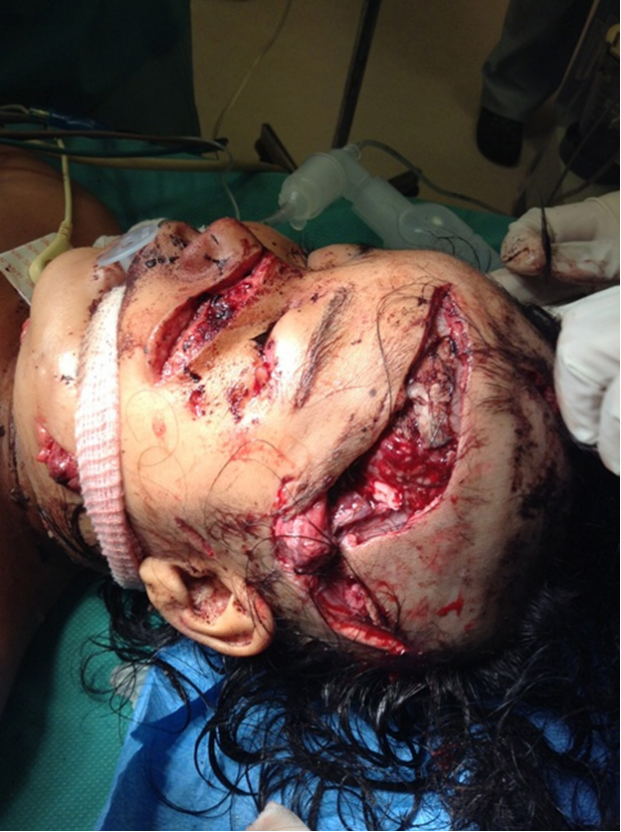
Child in the operating room.

About four hours post-arrival and nine hours post-injury, her vital signs were blood pressure 100/62mmHg, pulse 100/minute, temperature 99.8°F axillary, and respiratory rate 32/minute. At that point she was taken to the operating room. They found an open fracture to the left fronto-temporal region, a depressed fracture to the left middle parietal bone, and boney defects in the left frontal zygomatic arch and left frontal bone. A depressed fracture of the left posterior parietal bone with a 5cm dural tear was flushed with normal saline and the edges debrided and skin closed (Figure 4). An open fracture to left angle of mandible was washed and closed, and a laceration to the left nostril extending onto the left side of the face was repaired.

**Figure 5:**
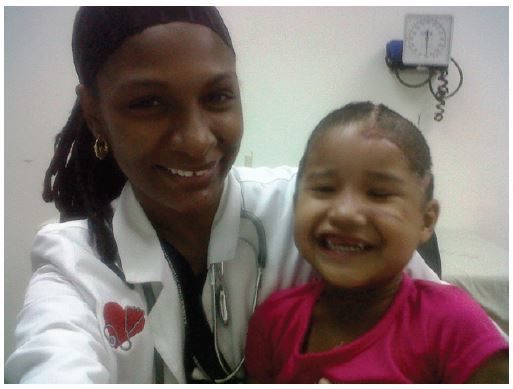
Child in the clinic prior to discharge (published with written parental permission).

Post-operatively, the child was sent to the intensive care unit on a ventilator. She received two units of packed erythrocytes—one intra-operatively and one post-operatively. Post-operative laboratory results were: Hgb 9.3g/dl, WBC: 11,500/mm^3^ (polys 62%, lymphs 30%, bands 8%), platelets 171,000/μL, Na 136mEq/L, K 4.4mEq/L, Cl 109mEq/L, BUN 12mg/dL, creatinine 0.6 μmol/L. Post-operatively, she received metronidazole, amoxicillin/clavulanate, and gentamycin. The child self-extubated on the third day and was discharged the next day to the pediatric surgical ward. She was discharged from the hospital 22 days after arrival (Figure 5).

The child returned home to normal activities. Her only deficits are healing wounds with some scarring across her face and a left ptosis, most probably from local nerve injury.

## DISCUSSION

This case describes a severe life-threatening attack of a non-captive jaguar on a human child in South America, which has been rarely described in the medical literature.^1^ The jaguar evolved in Europe or Asia at least 1.8–2.0 million years ago and colonized the Americas via the Bering Strait and the Panama (Darien) Isthmus. Although it looks much like a leopard (*felis pardis*), DNA studies indicate that it is more closely related to the lion (*P. leo*) or the snow leopard (*P. uncial*).^6^ The word “jaguar” comes from one of the Tupi–Guarani languages, and means all carnivorous beasts. Their specific word for jaguar is *jaguareté*, with the suffix -*eté* meaning “true.”^7^

### Geographical Distribution

Jaguars currently inhabit a region from northern Argentina to as far north as southern Arizona, a ranging area that has contracted approximately 46% over the past century.^8^ The child in this case lives within the Guyana-Montane Forest, where the numbers of jaguars have decreased over the last decade.^9^,^10^ While the jaguar prefers dense rainforest, it will range across a variety of forested and open terrains. It is strongly associated with the presence of water and is notable, along with the tiger, as a feline that enjoys swimming.^11^ The child in this case was attacked adjacent to a major waterway.

### Population Decline and Protection

A jaguar’s typical lifespan in the wild is around 12 to 15 years; in captivity they live up to 23 years.^11^ Human population growth inevitably leads to jaguar population decline and eventual local extinction. This stems from deforestation and fragmentation of their habitat with barriers.^6^ Both are occurring in the region in which this attack occurred. Jaguars also are killed by ranchers and farmers to protect their livestock or out of fear, and by professional hunters for their skin and other body parts.^6^ The jaguar is considered “near threatened”^12^ or “endangered,”^13^ and all commercial trade is prohibited.^14^

### Anatomy

The jaguar has a short and sturdy physique that makes it adept at climbing, crawling, and swimming.^11^ It normally weighs 56 to 96kg (124 to 211lb), with larger males weighing as much as 160kg (350lb) (roughly matching a tigress or lioness). Females are typically 10% to 20% smaller than males.^15^ Further north, its size diminishes. In Guyana, specimens up to 200lb have been seen, while in the forests of Paraguay, Southern Brazil and Bolivia, jaguars weigh up to 300lbs. The jaguar is more powerfully and heavily built than the leopard, standing 63 to 76cm (25 to 30in) tall at the shoulders. Their length, from the nose to the base of the tail, varies from 1.2 to 1.95m (3.9 to 6.4ft).^15^

Jaguars have sharp, strong, retractile claws, which they use to grasp prey and puncture its spine, cervical soft tissues, and skull. Deep lacerations and tissue loss from their claws also often occur. Wounds have included extensive deep lacerations of cervical structures, including transection of the trachea, and lacerations of the jugular vein, carotid artery and cervical nerves. Some of these injuries were only discovered during operative exploration.^16^ In other cases, cervical spine dislocations and spinal cord transections were only discovered at autopsy.^1^,^17^ Most of this patient’s injuries were from clawing, rather than from bites, although her potentially lethal wound was the bite through her skull.

Jaguars generally have a tawny yellow coat, but it can range from reddish-brown to black. The dorsal coat is covered in rosettes for camouflage and its ventral area is white. The spots vary over individual coats and between individual jaguars: rosettes may include one or several dots, and the shapes of the dots vary. The spots on the head and neck are generally solid, as are those on the tail, where they may merge to form a band.^11^ The rosettes on a jaguar’s coat are larger, fewer in number, usually darker, and have thicker lines than the leopard, as well as small spots in the middle that the leopard lacks. About 6% of jaguars are black (melanistic) “panthers,” a designation that stems from polymorphism rather than denoting a separate species.^11^ They reside, among other locations, along the Guyana–Venezuelan border, where the described attack occurred.^6^

### Hunting

Jaguars are opportunistic killers, walking until they encounter prey and eating what is available. Jaguars’ hunting activity varies with prey availability, which means it hunts mostly at night. They have very large (males 28–40km;^2^ females ≥10km^2^) non-overlapping hunting ranges, often changing the specific hunting area within their range every two weeks.^18^ That makes two attacks in the same area on the same child within a month extremely unusual. On average, a jaguar kills a large- or medium-sized prey every four days, although they typically do not consume an entire large carcass.^6^ In captivity, a 76kg jaguar typically consumes the equivalent of 1.4kg/day (34g/day/kg cat).^19^

The jaguar tends to take larger prey, usually over 22kg (49lb), and often weighing 10%–80% of its own body mass.^19^ Their preferred natural prey includes capybara (*Hydrochoerus hydrochaeris*, a large aquatic rodent weighing ~25kg), peccaries (*Pecari tajacu* and *Tayassu peccary*), tapirs (*Tapirus terrestris*), and caimans (Alligatoridae family).^1^ When it encroaches on domestic animals, “it is by no means an unusual feat for a 150lb jaguar to kill a 1,000lb steer, and drag the carcass for a hundred yards to the seclusion of some thicket and there to dine in comfort.”^15^

### Children as Prey

Since the size of their prey influences large cats, children make especially likely targets for attacks.^3^ In the United States, the majority of big cat attacks involve children.^3^ The child in this case closely matched the physical characteristics of a jaguar’s typical prey. Not only the child’s size and weight (24kg), but also her head size was very similar to that of capybara (average skull length 18–20cm).^20^

### Killing Behavior

Jaguars stalk and ambush, rather than chase their prey, attacking from nearby cover and quickly pouncing from a target’s blind spot—sometimes from a tree.^15^ With bigger prey they may jump over them, biting the nape of their neck and dragging them down.^6^ The ambush may include leaping into the water after its prey or, as in one reported case, knocking its human victim out of a boat, since a jaguar is quite capable of carrying a large kill while swimming.^1^

The jaguar skull is robust and massive, supporting a powerful jaw with very long and massive canines. Among mammals, the jaguar has the third most powerful bite, estimated at 74% of a *P. leo* (lion) bite force and 84% of a *P. tigris* (tiger)—both of which have an estimated biting force of more than 1,000lb/square inch.^21^,^22^ This allows it to pierce the shells of armored reptiles and to directly bite through a turtle’s shell or a tapir or cow’s skull, penetrating its brain.^6^ According to one scientific observer, “The jaguar seems to take the head into its mouth and with an opposing set of canines bite one or more times until the teeth penetrate the brain.”^20^ That was the most serious injury our patient received.

## EVALUATION AND TREATMENT

This patient’s evaluation and treatment reflects other cases described in resource-limited settings. It parallels, but does not exactly replicate, recommended optimal treatment for these injuries (Figure 2). After the child was attacked in a remote area at the margin of the Amazon Basin, relatives and neighbors forced the jaguar to drop the child and then killed the animal. They then transported the child to the local nurse-staffed health center, where the nurse administered the most appropriate medications she had available and washed the wounds with water and applied moist dressings, most of which the child removed. She wanted to use povidone-iodine as a potential antiviral agent against rabies, but none was available.^23^ The possibility of rabies existed after this unprovoked attack by a wild mammal that has been known to carry rabies.^24^ She assumed (correctly) that the child had received primary tetanus immunizations and so did not administer it.

The child’s relatively short transport time from an almost inaccessible jungle area to the country’s main hospital was a remarkable feat. The child’s village is located eight hours (by jetboat) and two weeks (by motorboat) up river from the nearest town (Bartica, Essequibo). The ready availability of medical evacuation is in part due to the Guyanese government guaranteeing payment for such flights.

The child arrived at the ED unannounced, since no mechanism exists for contacting hospitals from remote regions—either for guidance or to inform them about arriving patients. No telephone service exists anywhere near the child’s village. She was transported on her mother’s lap via a small fixed-wing plane, since no other means were available. A cervical collar was applied only upon her arrival at our ED. After arrival in the tertiary care facility ED, the evaluation and treatment closely followed the steps in Figure 2.

A careful primary and secondary trauma-oriented exam demonstrated that the neck and torso wounds were not deep enough to have caused structural damage, while the facial wounds communicated with bony fractures and the scalp wound contained an obvious (when examined carefully) puncture wound of the skull. We started IV lines with 0.9% normal saline, drew bloods for baseline laboratory tests and type and cross-match, further cleaned the wounds and administered antibiotics.

Since the child appeared clinically stable—and neurologically normal – and our chest radiograph and eFAST exam were negative, we agreed with the consulting surgeons that it was safe to send the child to a nearby institution for a CT of her head and cervical spine, since the scanner associated with the hospital (but which still would have required a short ambulance transfer) was temporarily inoperable. We discussed administering rabies post-exposure prophylaxis for this unprovoked attack of a mammal in a rabies-endemic area; however, none was available.

Given the extent of soft tissue and intracranial damage, the child was taken to the operating room for a craniotomy and facial bone and soft tissue debridement and repair. While mammalian bite wounds carry an infection rate of 10%–20%, all her wounds were closed primarily, since most were on her face and scalp, they were highly vascular and she appeared to have few of the major risk factors for infection: 1) devitalized or low-vascularity areas; 2) deep punctures, macerated or crushed tissue; 3) tissue loss or avulsion; or 4) patients >50 years old who have chronic diseases or are immunocompromised.^1^,^25^

The patient received transfusions to bring her Hgb levels above 8g/dL. Her antibiotics were changed to match the polymicrobial organisms anticipated from these injuries. Mechanical ventilation continued until self-extubation, an unfortunate but relatively common occurrence in ICUs with limited staff. After discharge from the hospital, she and her relatives stayed in an Amerindian housing unit supplied by the government. During her subsequent clinic visit, she appeared to be doing well, so she and her family returned to the jungle. Arrangements were made for her to be seen at our hospital again in several months, at which time she again would be transported by a government-arranged plane.

## CONCLUSION

Jaguar attacks on humans have increased as their habitat and available prey decreases and humans come in close contact with them more frequently. Clinicians assessing and treating these injuries must be aware of three critical and possibly occult injuries: disruption of the cervical spine, which may cause immediate death; intracranial tooth penetration; and laceration of the large cervical vessels. Awareness of these injuries, especially occult intracranial penetration, may help clinicians better manage these patients. In the remote areas where these attacks often occur, however, evaluation and treatment must succumb to optimizing the available resources.
